# Efficient Image Retrieval Using Hierarchical K-Means Clustering

**DOI:** 10.3390/s24082401

**Published:** 2024-04-09

**Authors:** Dayoung Park, Youngbae Hwang

**Affiliations:** Department of Control and Robot Engineering, Chungbuk National University, Cheongju 28644, Republic of Korea; wnidy100@cbnu.ac.kr

**Keywords:** image retrieval, CBIR, efficiency, hierarchical clustering, tree search

## Abstract

The objective of content-based image retrieval (CBIR) is to locate samples from a database that are akin to a query, relying on the content embedded within the images. A contemporary strategy involves calculating the similarity between compact vectors by encoding both the query and the database images as global descriptors. In this work, we propose an image retrieval method by using hierarchical K-means clustering to efficiently organize the image descriptors within the database, which aims to optimize the subsequent retrieval process. Then, we compute the similarity between the descriptor set within the leaf nodes and the query descriptor to rank them accordingly. Three tree search algorithms are presented to enable a trade-off between search accuracy and speed that allows for substantial gains at the expense of a slightly reduced retrieval accuracy. Our proposed method demonstrates enhancement in image retrieval speed when applied to the CLIP-based model, UNICOM, designed for category-level retrieval, as well as the CNN-based R-GeM model, tailored for particular object retrieval by validating its effectiveness across various domains and backbones. We achieve an 18-times speed improvement while preserving over 99% accuracy when applied to the In-Shop dataset, the largest dataset in the experiments.

## 1. Introduction

The modern field of CBIR is categorized into particular object retrieval and category-level retrieval [1,2,3,4,5]. In recent CBIR systems, images are typically represented as global feature vectors, and inter-vector distance metrics such as cosine [3] and Euclidean [6] distances are used to determine the similarity between a query and the database. Extensive studies on CBIR have been conducted on backbone networks utilizing various deep learning models, including convolutional neural networks (CNN) [2,3,7,8,9,10,11,12], vision transformers (ViT) [5,13,14,15], and graph convolutional networks (GCN) [16,17], as encoders for image vector representations.

One of the significant challenges in the image retrieval task is the retrieval time and computational cost. If the similarity between database and query descriptors is calculated without the use of any indexing technology, the time and computational resources required can become prohibitive, especially when dealing with large-scale datasets such as those found on the Internet, where the number of stored image samples is increasing exponentially, including video materials consisting of frame-by-frame images. Therefore, it is essential to employ an appropriate database classification or an indexing scheme for the practical application of image retrieval systems in real-world scenarios. Particularly in applications that demand real-time image retrieval, such as visual localization, where camera sensors are used to estimate location, the reduction of retrieval speed becomes a critical priority. Research focused on enhancing image retrieval accuracy is a dynamic and diverse field [4,18]. In contrast, studies aimed at improving retrieval speed show a less dynamic trend, with recent research primarily leaning towards hashing-based techniques [4,19,20,21].

Recognizing the need for progress in accelerating image retrieval, we explored the development of an auxiliary module that aligns with existing configurations of capable backbone networks, considering that several high-performance approaches have already been proposed. Following this exploration, our investigation focused on applying tree indexing techniques based on hierarchical K-means clustering, traditionally used for local descriptors, to global descriptors, which yielded promising results.

Our method is inspired by the approach of vocabulary tree [22], which performs hierarchical K-means clustering on local feature vectors and scores them according to inverse document frequency weighting. It can also be applicable to deep neural network-based global descriptors for reducing retrieval time. In this paper, we divide the process related to tree construction and search into two stages, namely, offline and online steps. In the offline step, we extract database descriptors and cluster them hierarchically. The online step involves identifying similar leaf nodes within the pre-generated cluster tree and extracting target images by computing and ranking the similarity between the query and the database. We introduce three tree search algorithms that can be used selectively depending on the importance between retrieval speed and accuracy, which has a trade-off relationship.

**Figure 1:**
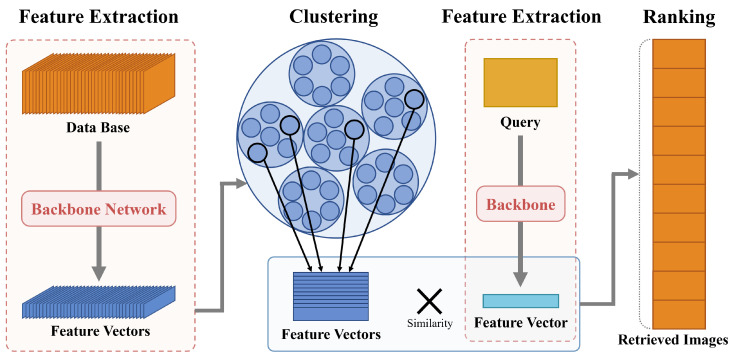
The framework of the image retrieval system employing the proposed method.

The framework of the proposed image retrieval is shown as Figure 1. It offers flexibility, making it applicable to numerous content-based image retrieval (CBIR) methods and compatible with various backbone networks designed for vector representation. Based on the tree search algorithm that yields the best retrieval accuracy, our method achieves speedups of 5, 6, and 18 times on the category-level retrieval datasets CUB, CARS, and In-Shop, respectively, when combined with the existing CLIP-based model. When integrated with the established CNN-based model, our method achieves speedups ranging from 2 to 3.5 times for the particular object retrieval datasets Oxford, Paris, ROxford, and RParis, respectively.

To summarize, our contributions include the following:

- We significantly improve the speed of the online step of CBIR by hierarchically indexing the global deep features of the database using hierarchical k-means clustering.
- We propose three tree search methods, intensive search, relaxed search, and auto-search, each of which is a trade-off between accuracy and speed of CBIR so that you can choose the one that fits your performance needs.
- We apply the proposed method to UNICOM, a category-level CBIR model that has achieved the state-of-the-art, and show a reduction rate of 99.5% for retrieval time. By applying it to R-GeM, a CNN-based particular object CBIR model, we show a speedup of 71.8%.

## 2. Related Work

The goal of CBIR is to find images in a database that contain content similar to a given query image. The process of performing CBIR can be roughly divided into two steps: feature extraction and search process [21,23,24]. The feature extraction step extracts image features that allow for a comparison between the database and a query image to measure similarity, and the performance of CBIR has been heavily influenced by advances in various deep learning models. Traditional feature extraction techniques are mainly based on local feature extractors, which were dominant in the 2000s [18]. A typical local feature extractor, SIFT [25], uses image pyramids to extract size and rotation-invariant features. Another well-known algorithm, SURF [26], improves the slow detection speed of SIFT by varying the size of the filter instead of the image pyramid. CBIR models using these local feature extraction algorithms are characterized by their robustness to various environmental variations such as scale, perspective, rotation, and illumination [27,28,29,30,31,32]. Bag of visual words (BoVW) [33], derived from the concept of bag of words (BoW) [34] in natural language processing (NLP), uses a histogram of salient features to represent an image as a single vector, which makes it possible to combine multiple feature extractors, resulting in better image retrieval performance than using a single model [31].

Recent studies on image retrieval have been dominated by deep learning-based methods. In particular, the development of CNNs has been instrumental in significantly improving the performance of image retrieval by effectively representing images as compact vectors [21]. The simplest way to utilize CNNs is to use pre-trained off-the-shelf models as encoders for image classification tasks [2,7,8,9,10]. In more time-consuming approaches, CNN models are fine-tuned to obtain feature extraction capabilities that are better suited to new datasets [3,11]. As a result, global descriptors are generated from the output of the convolutional layer, either through a fully connected (FC) layer [12] or through a global pooling method. Typical global descriptors include maximum activation of convolutions (MAC) [2], sum pooling of convolutions (SPoC) [8], or generalized mean pooling (GeM) [3]. In particular, GeM, which is a trainable method, improves search performance by generalizing SPoC and MAC. The global descriptor combination framework proposed by [35] is end-to-end trainable and efficient compared to other ensemble techniques, while outperforming each global descriptor separately.

More recently, convolution-free models, where the transformer layer replaces the traditional convolutional layer, have demonstrated superior performance on image retrieval tasks. Following experiments using off-the-shelf ViT [13], methods to fine-tune them for image retrieval tasks have been attempted [5]. Deep token pooling (DToP), proposed by [15], utilizes a structure comprising a CNN stem and a ViT encoder, making it the first transformer-based method to surpass CNN and achieve state-of-the-art performance in particular object retrieval. The study collects global and local features from multiple layers of classification and patch tokens, respectively, and then enhances the locality of interactions through multi-scale convolution.

Contrastive language-image pre-training (CLIP) [36] is a potent multi-modal representation learning model trained on LAION 400M, an extensive dataset which contains a large-scale collection of image–text pairs. This model has gathered significant attention in current research efforts, finding applications in various computer vision tasks, including image generation [37], object detection [38], face recognition [39], semantic segmentation [40], and image retrieval [6,41]. [6] proposes a CLIP-based cluster discrimination method that goes beyond CLIP and OPEN-CLIP [42] in both classification and zero-shot image retrieval tasks. In the clustering step, it combines each image–text feature pair produced by the pre-trained CLIP and performs multi-modal K-means clustering on those features. In the discriminative step, it applies both random class selection and random feature selection to enhance the compactness of features while minimizing the probability of inter-class conflict. This model was used as a baseline for the experiments in this study.

In CBIR, both image representation and computational cost are essential considerations. As the size of the dataset increases, the computational cost and time inevitably increase with the general retrieval method of computing the similarity between descriptors. Therefore, various indexing structures and schemes have been proposed to make the retrieval task efficient. Among them, hashing-based methods have received much attention due to their great efficiency in computation and storage. By projecting high-dimensional features into a low-dimensional space, generating a compact binary code, and using binary pattern matching or hamming distance measurement, binary hashing can achieve search speed improvement and computational cost reduction [19,20].

There are also studies on various tree-based methods. [43] proposed a parallel indexing scheme based on multiple descriptor trees to facilitate the evaluation of image content descriptors. The tree is a variant of the in-memory BK-tree [44], with an arbitrary number of children representing the distance between the node and its parent. [22] proposed an image retrieval system that utilizes a vocabulary tree generated through hierarchical K-means clustering. To generate a vocabulary tree, local feature vectors via MSER [45] and SIFT are first extracted from the training dataset, and then K-means clustering is performed to classify them into K branches. Then, this process is recursively applied to each branch until a specified maximum L-layer is reached, resulting in K sub-branches at the next level. Once the tree is prepared, during image retrieval, each feature vector of the query image is compared layer-by-layer with the K candidate class centers, and the most similar class is selected. Finally, the image containing the most features within the selected class is extracted. Here, the similarity to each class is determined using the term frequency–inverse document frequency (TF-IDF) scoring method.

There are other studies based on hierarchical k-means clustering using local features. Murphy [46] performs hierarchical k-means clustering on MSER feature vectors and then extracts the database images with the k feature vectors closest to the query using k-NN to extract a database image such that the most feature vectors are in the nearest neighbors. In [47], a wavelet-based global color histogram is used as the database, and Euclidean distance is used to find and rank similar data. Mantena and Anguera [48] present a study in the field of audio processing that performed node traversal in a cluster tree of audio reference data based on the distance of the centroid vector from the query to the maximum radius of the cluster. To perform large-scale video retrieval, Liao et al. [49] performs sample-based hierarchical adaptive k-means clustering on MIFT [50] descriptors of video key frames to generate visual vocabularies, and it performs tree search based on Euclidean distance. Instead of using local features in the above studies, we apply hierarchical k-means clustering to *deep global features* for efficient image retrieval, more specifically, category-level and particular object CBIR applications. Furthermore, we additionally propose three search methods by balancing between accuracy and speed.

## 3. The Proposed Method

This section presents an approach to use hierarchical K-means clustering of global descriptors for image retrieval acceleration. This methodology consists of two stages: the first stage is an offline step, focused on extracting descriptors from the database and constructing a descriptor tree. The second stage is an online step, which utilizes a tree search to filter out similar candidate descriptors in the database. Subsequently, this online step ranks the descriptors of the database based on the computed similarity.

### 3.1. Offline Step

Before initiating the image retrieval process for a given query image, we begin by extracting the global descriptors of each image within the database using the backbone network. These descriptors are served as the basis for subsequent hierarchical K-means clustering, which is established based on the similarity among the descriptors. It is worth noting that most models capable of representing an image as a compact vector can be served as the descriptor extractor.

**Figure 2:**
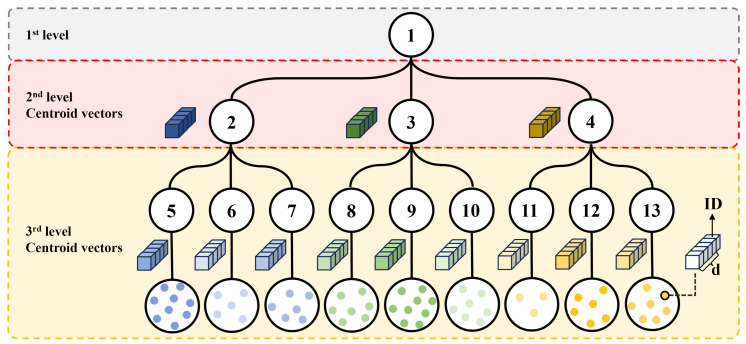
Structure of the tree generated in the offline step. This is an example for k=3, l=3. *d* is the size of a database descriptor.

The hierarchical clustering of the extracted embeddings results in a tree structure, as depicted in Figure 2. In Figure 2, an example is provided with a tree height of l=3 and each node having k=3 child nodes. Both of these values are hyperparameters. For a tree of height *l*, the total number of nodes in the tree for each level *m*, 1≤m≤l, is $k^{m-1}$. Furthermore, the total number of internal nodes in the tree is $I=\frac{k^{l-1}-1}{k-1}$. In the process of generating a tree structure, each node is indexed in ascending order, starting from the top and advancing from left to right, with the initial node indexed as 1. The set of descriptors for all database images is initially stored at the first node. Subsequently, we execute the k-means clustering procedure using the descriptors at the root node. Then, the centroid vectors of the *k* derived clusters and their image descriptors are stored in each of the *k* child nodes. Furthermore, for memory optimization, the image descriptors initially stored at the root node are all removed. This process is recursively performed at each node up to the (l−1)-th level. As a result, all nodes, except for the root node, store centroid vectors that represent their respective clusters. The set of descriptors for database images is specifically stored in the leaf nodes. In other words, counting the number of descriptors within all leaf nodes yields the total number of images in the database.

(1) $$\underset{C^{n}}{argmin}\sum_{i=1}^{K}\sum_{e_{j}\in C_{i}^{n}}\Phi \left(e_{j},\mu_{i}^{n}\right),\;n=1,2,\cdots ,I$$

where $\mu_{i}^{n}$ and $C_{i}^{n}$ denote the centroid vector and the set descriptors belonging to the *i*-th cluster derived from the descriptors stored at node *n*, respectively. The function Φ is a user-specified vector distance metric. Notably, the tree generation process, with parameters k=3 and l=4, is accomplished in just 2.8 s on a single GPU, utilizing 12,612 images.

### 3.2. Online Step

In the online step, our work focuses on tree search methods, and we present three tree search algorithms. The common objective of these algorithms is to traverse the tree based on the distance between a query descriptor and a centroid vector stored at each node, extracting only some of the most similar leaf nodes from the tree generated during the offline step. Following the selection of the final leaf nodes, proceed to calculate the distance to the query descriptor for each database descriptor within these selected nodes. Finally, rank them in order of closest distance. The proposed three algorithms exhibit distinct variations in retrieval accuracy retention and speedup rates, allowing for the selective application of one of the three algorithms based on the performance requirements of your task.

The intensive search method selects only one node per level in the tree, leading to the identification of a single-leaf node; while it is the fastest approach, its accuracy might be compromised compared to the others. On the other hand, the relaxed search approach allows for the selection of more than one node per level, providing a more flexible node selection; while this algorithm has the best ability to preserve the retrieval accuracy of the previous model that has not applied the proposed method, it is the slowest of the three methods. The degree of relaxation can be adjusted through two additional hyperparameters. Lastly, the auto search algorithm selects multiple nodes per level like relaxed search but does not require any additional hyperparameters. The retrieval accuracy and speed when using this algorithm are positioned in between the previous two tree search algorithms. Here, we define the index of the leftmost node within the *m*-th level as $f\left(m\right)=\frac{k^{m-1}-1}{k-1}+1$. In this section, we define a query embedding as $e_{q}$, the set of indices for each node within the *m*-th level as $H^{m}={\left\{i\right\}}_{i=f(m)}^{i+k^{m-1}-1}$, and the set of centroid vectors for each node *i* within the *m*-th level as $\Psi^{m}=\left\{\mu_{i}\;|\;i\in H^{m}\right\}$.

#### 3.2.1. Intensive Search

An intensive search algorithm is designed to prioritize speed in the search process. This algorithm explores the tree from top to bottom, identifying the closest node to the query at each level based on the distance between the query descriptor and the centroid vector stored at each node. At each level, all children nodes of the unselected nodes are excluded in real time during the tree search process. The required operations for tree search include solely the computation of similarity between a k×d size matrix per level of the tree and a *d*-size vector, along with the sorting of *k* values. The aim of the intensive search is to pinpoint a single leaf node that bears the closest resemblance to the query descriptor. While it excels in terms of rapid execution, the algorithm’s emphasis on speed may lead to a potential compromise in accuracy when compared to other, more meticulous search approaches. Despite its potential trade-offs, the intensive search can serve as an important role in scenarios where immediate responses are of utmost importance, providing a valuable balance between timely processing and acceptable accuracy.

#### 3.2.2. Relaxed Search

The relaxed search algorithm, in contrast to the intensive search, uses a more flexible and comprehensive approach during the tree traversal process. By allowing the selection of more than one node per level, the relaxed search yields a more comprehensive selection of leaf nodes that are similar to the query descriptor. Let $N^{m}$ denote the set of node indices selected at the *m*-th level, i.e., those considered for exploration. Initially, *N* is always initialized to N=H. Then, at each level where m≥2, update $N^{m}$ and $N^{m+1}$ after completing the node selection. Since the first level of the tree contains only a single node, it is excluded from the node selection process. The nodes to be selected at each level satisfy one or more of the following two conditions:

(2) $$N^{\prime }=\left\{i\;|\;\Phi \left(\mu_{i},e_{q}\right)\leq s_{1},\;i\in N^{m}\right\}$$

(3) $$N^{\prime \prime }=\left\{i\;|\;\Phi \left(\mu_{i},e_{q}\right)-\underset{j\in N^{m}}{min}\Phi \left(\mu_{j},e_{q}\right)\leq s_{2},\;i\in N^{m}\right\}.$$

$N^{m}$ is updated with all the nodes extracted in Equation (2) or Equation (3) as follows:

(4) $$N^{m}\leftarrow N^{\prime }\cup N^{\prime \prime },\;2\leq m\leq l.$$

Subsequently, $N^{m+1}$ is updated with the set comprising all child nodes of the nodes included in the updated $N^{m}$ as follows:

(5) $$N^{m+1}\leftarrow \left\{i\;|\;P\left(i\right)\in N^{m},\;i\in N^{m+1}\right\},\;2\leq m\leq l-1$$

where P(i) is a function that returns the index of the parent node for node *i*. Equation (5) involves removing all child nodes of the nodes dropped at level *m*. Since leaf nodes have no children, the update of $N^{m+1}$ carried out at the *m*-th level is performed only up to the m=l−1 level, not m=l. Repeat this process of updating N until m=l, and ultimately, $N^{l}$ will encompass the indices of the most similar leaf nodes. This algorithm allows for the selection of multiple nodes per level in the tree, enabling a broader exploration of the available descriptors. Because of the broader exploration, the relaxed search operates at a relatively slower pace compared to the other two methods, counterbalanced by the algorithm’s ability to preserve an existing retrieval accuracy almost identically. The degree of relaxation within the search process can be fine-tuned through two hyperparameters, $s_{1}$ and $s_{2}$, providing additional control over the extent of exploration and the resulting retrieval accuracy.

#### 3.2.3. Auto Search

The auto search algorithm is designed to automate the process of determining the optimal value for the hyperparameter $s_{1}$, with a focus on Equation (2), which plays a pivotal role in the node selection process of the relaxed search. Additionally, this method eliminates the need for Equation (3), further streamlining the computational process. Consequently, auto search proves to be more efficient and less time-consuming in the online step compared to relaxed search. In contrast to relaxed search, this approach sorts the distances between the centroid vectors of each node in $N^{m}$ and the query descriptor in real time at each *m*-th layer, arranging them in ascending order, and defining it as $D^{m}$ as follows:

(6) $$D^{m}={\left\{\Phi (\mu_{j},e_{q})\right\}}_{j\in N^{m}},\;D_{1}^{m}\leq D_{2}^{m}\leq \cdots \leq D_{\left|N^{m}\right|}^{m}$$

where $D_{i}^{m}$ represents the *i*-th element of $D^{m}$. Then, for the ordered set of distance values $D^{m}$, identify the node with the largest difference from its neighbor, and set $s_{1}$ to the distance value of that node as follows:

(7) $$s_{1}=\underset{\theta_{j}}{argmax}\left(\theta_{j+1}-\theta_{j}\right),\;\theta_{j}\in D^{m},\;1\leq j<\left|N^{m}\right|$$

and update $N^{m}$:

(8) $$N^{m}\leftarrow \left\{i\;|\;\Phi \left(\mu_{i},e_{q}\right)\leq s_{1},\;i\in N^{m}\right\},\;2\leq m\leq l.$$

Finally, as with the relaxed search, update $N^{m+1}$ using Equation (5). This algorithm automatically adjusts the distance threshold for defining similar nodes, eliminating the need for manual intervention while still demonstrating retrieval accuracy close to that of relaxed search. Consequently, the speed is slower than the intensive search, yet faster than the relaxed search, while the retrieval accuracy is superior to the intensive search but slightly inferior to the relaxed search.

## 4. Experiments

### 4.1. Datasets

We evaluate our proposed method using various datasets in two categories: category-level retrieval and particular object retrieval. The datasets used for the evaluation include CUB [51], consisting of 11,788 images representing 200 distinct bird categories, CARS [52], comprising 8131 images categorized into 98 different car types, and In-Shop [53], a set of 72,712 clothing item images categorized into 7982 groups, with 3997 categories designated for training and the remaining 3985 categories partitioned into 14,218 queries and 12,612 gallery images for testing. The Recall@1 is computed to assess the performance of category-level retrieval.

For particular object retrieval, our analysis focuses on four datasets: Oxford, Paris [54,55], ROxford, and RParis [56]. Oxford contains 5063 images distributed across 11 categories, with 55 associated queries. Similarly, Paris includes 6392 images divided into 11 classes, with 55 queries provided for assessment. ROxford and RParis are revisited benchmarks of Oxford and Paris, respectively. ROxford comprises 4993 images distributed among 13 categories, with 70 corresponding queries. On the other hand, RParis includes 6322 images categorized into 12 classes, along with 70 queries for evaluation. The performance evaluation for these datasets is conducted using the mean average precision (mAP). Specifically for the revisited benchmarks, the evaluation is further stratified into three levels of query difficulty: easy, medium, and hard.

These datasets have been carefully selected for the comprehensive evaluation and validation of our proposed image retrieval framework, demonstrating their effectiveness across various image retrieval tasks. The details of the datasets utilized in our experiments are summarized in Table 1.

### 4.2. Implementation Details

In our experiments, we used the ViT L/14-336 UNICOM, pre-trained on the LAION 400 M image-text dataset, as our baseline. All experiments using UNICOM, a CLIP-based model, followed the evaluation setup for zero-shot image retrieval described in the original paper [6]. Furthermore, to verify the generality of the proposed framework, we conducted experiments using R-GeM [3], a CNN-based model for image retrieval, as the backbone network. All experiments were conducted on an Intel(R) Core(TM) i7-10700 CPU @ 2.90 GHz with 8 cores (Santa Clara, CA, USA). Unless stated otherwise, the default hyperparameter settings were k=3, l=4, $s_{1}=1$, and $s_{2}=0.16$ for the entire experiments. The evaluation datasets encompassed both domains, category-level retrieval, and particular object retrieval. Through this composition of datasets, we demonstrated the applicability of our framework to both major domains of CBIR.

### 4.3. Comparisons to Baseline Models

#### 4.3.1. Category-Level Retrieval

We evaluated the proposed framework on the category-level retrieval datasets CUB, CARS, and In-Shop. The retrieval performance for each dataset is represented by Recall@1, following the evaluation methodology of the existing study on UNICOM. Recall@1 is measured by calculating the percentage of queries where the top-ranked image is positive. Specifically, it is the number of times the top-ranked image in the retrieval result is a true positive, divided by the total number of queries. Therefore, higher values of Recall@1 indicate that the system is more adept at accurately retrieving positive images, thus demonstrating superior performance.

Table 2 demonstrates the impact of the proposed method on the image retrieval task when applied to UNICOM. In the table, the model that combines the proposed method is denoted as UNICOM+Ours, with Intensive, Auto, and Relaxed indicating when intensive, auto, and relaxed searches are used in tree search, respectively. The table includes Recall@1, number of operands, and retrieval time. It also specifies the Recall@1 retention, operand reduction, and search time reduction as a percentage compared to UNICOM. The number of operands is the number of the database images contained in the leaf nodes finally selected in the tree search. Image retrieval time is measured in milliseconds and represents the execution time of the online step excluding the offline step.

The speedup is observed to be higher in the order of intensive, auto, and relaxed searches, while the retention of retrieval accuracy follows the opposite pattern. In the case of the CUB dataset, a slight improvement of approximately 0.1% in accuracy is observed when utilizing relaxed search, suggesting a potential for performance enhancement depending on the clustering results. The difference between the reduction rate of the number of operands and the retrieval time is greater in the order of intensive, auto, and relaxed searches due to the overhead incurred during the tree search algorithm. The speedup tends to increase with the size of the database. As the size of the database increases, the speedup when using the intensive search algorithm is accompanied by a significant loss of retrieval accuracy that cannot be ignored. Therefore, the decision to adopt intensive search, which is the fastest, should be made by considering the performance requirements of the image retrieval task, especially for large datasets. On the other hand, relaxed search algorithm has a stable Recall@1 retention rate of over 99.6%, so for tasks where maintaining the Recall@1 of the original model is important, relaxed search is a good choice. However, since there are two additional hyperparameters in this algorithm, we can use auto search algorithm without hyperparameters with a high level of stable accuracy retention.

#### 4.3.2. Particular Object Retrieval

To evaluate the performance of the proposed framework in a particular object retrieval task, we employed well-known datasets, ROxford, RParis, Oxford, and Paris. In this work, mAP was used as the performance evaluation metric, which is used for assessing the effectiveness of retrieval systems.

Table 3 shows the performance change achieved by applying the framework proposed in this paper. R-GeM is an image retrieval model that shows good performance in particular object retrieval tasks. It combines GeM with ResNet101 [57], a classification model based on CNN, as a backbone network, and uses cosine distance metric to calculate the distance between images. The subcategories (E), (M), and (H) of mAP represent retrieval results for samples that fall into the easy, medium, and hard categories, respectively. The number of operands and retrieval time, and their respective reduction rates, have the same meaning as in Table 2. Performing image retrieval on the given datasets is a highly sensitive task as it requires precise identification and retrieval of specific objects from random local areas within complex images. This sensitivity is further heightened in samples belonging to the hard category where detection is complicated by various interferences, including distortions and occlusions. In this experiment, we employ only the superior accuracy-preserving capability of relaxed search. After fine-tuning the $s_{1}$ and $s_{2}$, we set them to $s_{1}=-0.01$ and $s_{2}=0.01$.

The retention rate of mAP is over 92.5 for all cases, including the easy, medium, and hard subsets in the revisited dataset, with an overall average of 97.8. Particularly, in the case of the hard category of RParis, mAP increases by about 2%, indicating a potential increase in accuracy depending on the dataset. Furthermore, the reduction in retrieval time is more significant in the order of Paris, RParis, Oxford, and ROxford, which corresponds to the size of the datasets. In other words, the speedup in image retrieval increases as the dataset size grows. However, the reduction rate in operands does not strictly follow this order. We speculate that this is because the difference in the speedup gained by reducing operands is smaller than the speed decrease caused by overhead in the tree search process. Although these results are implemented in Python, adopting a lighter programming language could further alleviate computational overhead, leading to an even greater increase in retrieval speed.

### 4.4. Ablation Study

#### 4.4.1. Scale of the Cluster Tree

**Figure 3:**
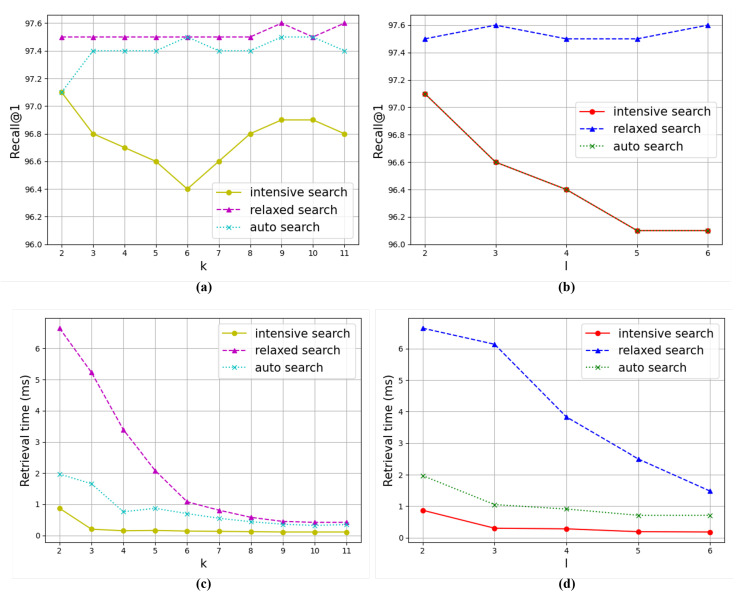
Evaluation of the model performance on the CUB dataset with respect to *k* and *l*. The employed model is a fusion of UNICOM and the proposed method, providing results for intensive search, relaxed search, and auto search. (**a**) Relationship between *k* and Recall@1. (**b**) Relationship between *l* and Recall@1. (**c**) Relationship between *k* and retrieval time. (**d**) Relationship between *l* and retrieval time.

Figure 3 illustrates the trends in image retrieval performance as the parameters *k* and *i* are varied. The analysis is conducted using the CARS dataset, and it encompasses measurements for the three tree search methods we introduced: intensive search, relaxed search, and auto search. In these experiments, we set l=2 for experiments with *k*, and k=2 for experiments with *l* to minimize the impact of non-experimental parameters on the results.

**Tree Width.** In the graph for parameter *k*, image retrieval accuracy and time follow the order of relaxed, auto, and intensive searches. In the case of intensive search, it achieves better accuracy with either small or large values of *k*, and it achieves the worst results when *k* has an intermediate value. Conversely, relaxed and auto searches demonstrate minimal fluctuations in accuracy as *k* varies, with relaxed search achieving better results and auto search achieving results as poorly as intensive search when k=2. Regarding retrieval time, all three algorithms display a pattern of decreasing time costs as *k* increases. The reduction is most pronounced in the order of relaxed, auto, and intensive searches, with relaxed search demonstrating an exponential decrease. Therefore, all three algorithms achieve the maximum temporal benefits when *k* has large values.

**Tree Height.** In the graph for parameter *l*, image retrieval times are higher in the order of relaxed, auto, and intensive searches. The same is true for retrieval accuracy, except that relaxed and auto search show the same graph, which differs from the graph for parameter *k*. The overlapping of the two graphs is attributed to the fixed value of *k* as 2. Consequently, within the auto search algorithm, the selection of nodes at each level narrows down to just one node, specifically the one that exhibits the highest similarity to the query, mirroring the behavior of intensive search. The accuracy of relaxed search is robust to changes in *l*, while auto search and intensive search tend to achieve worse results as *l* increases. In terms of retrieval time, the decrease is significant in the order of relaxed, auto, and intensive searches, especially for relaxed search. Therefore, relaxed search benefits from setting *l* to the maximum possible value, while the other two algorithms need to strike a balance between search accuracy and speed by adjusting *l* based on the required retrieval accuracy.

#### 4.4.2. Intensity of Relaxation

**Figure 4:**
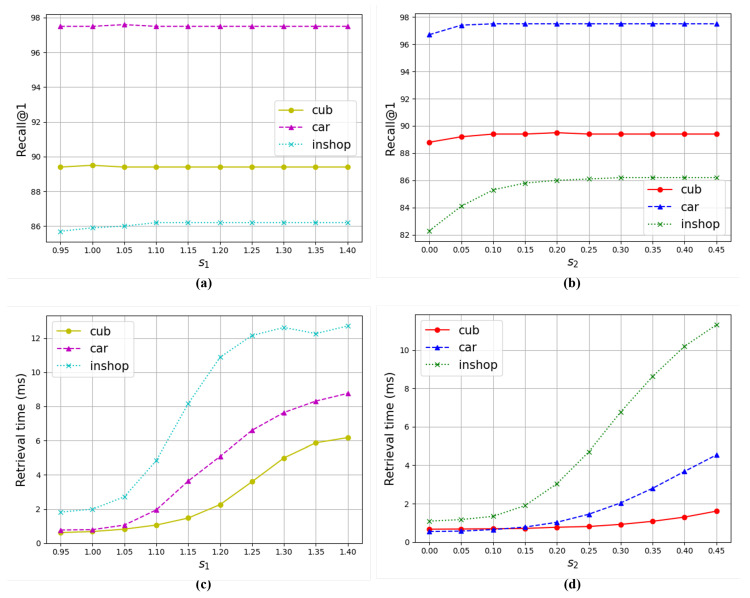
Evaluation of the model performance on the CUB dataset with respect to $s_{1}$ and $s_{2}$. The employed model is a fusion of UNICOM and the relaxed search version of the proposed method. (**a**) Relationship between $s_{1}$ and Recall@1. (**b**) Relationship between $s_{2}$ and Recall@1. (**c**) Relationship between $s_{1}$ and retrieval time. (**d**) Relationship between $s_{2}$ and retrieval time.

Figure 4 shows the image retrieval performance concerning two parameters $s_{1}$ and $s_{2}$ used in the proposed relaxed search algorithm. Here, we focus on analyzing the relationship between dataset size and hyperparameters by presenting experimental results on CUB, CARS, and In-Shop datasets, all of which have different sizes. All three datasets exhibit similar trends, with larger datasets displaying a more dramatic rate of change. The graphs for Recall@1 remain stable for both the $s_{1}$ and $s_{2}$ values, with a gradual decrease in accuracy below a certain value. This decline is more pronounced for $s_{2}$. The retrieval time for $s_{1}$ and $s_{2}$ continues to increase as it gets larger, but the rate of increase is amplified sharply at the point where Recall@1 starts to fluctuate. Therefore, for both $s_{1}$ and $s_{2}$, it is most efficient to use values close to the point where the retrieval accuracy begins to converge.

## 5. Conclusions

A key challenge in image retrieval has been the time and computational cost, especially for large-scale datasets. To address this, an auxiliary module is developed based on hierarchical K-means clustering. This method improves the practicality of high-accuracy methods by significantly reducing retrieval time while maintaining accuracy. The process includes an offline step for hierarchically clustering the database image descriptors using the K-means algorithm and an online step for extracting similarities and ranking them. Three tree search algorithms are introduced, catering to different speed and accuracy needs. To assess the performance of image category-level retrieval, we apply the proposed method to UNICOM for analyzing the effectiveness of the framework on various scales of datasets, including CUB, CARS, and In-Shop. According to the experimental results, among the three search strategies, intensive search shows the highest speed improvement, demonstrating retrieval time reduction rate of 97% or higher. Relaxed search maintains a stable accuracy retention rate of at least 99.7%. Additionally, auto search compromises the performance of both strategies, exhibiting a high speed improvement rate of over 80.1% in all cases. Furthermore, to evaluate the performance of particular object retrieval, we utilize R-GeM incorporating the proposed framework. Using the relaxed search, the results reveal a significant mAP retention rate of 92.5% to 102% and a high speed improvement rate ranging from 49.1% to 71.8%. Moreover, it is found that larger datasets resulted in significant reductions in image retrieval time.

In the real-world case, even though there will be a larger amount of classes and database images, our proposed method can be expected to achieve a higher speed based on these experiments. For practicality, we should consider rebuilding the cluster tree when new data come in, and the improvement of clustering performance is also important to increase retrieval accuracy.

## Figures and Tables

**Table 1:** Composition of datasets for evaluation of CBIR. The upper section, above the dashed line, represents the category-level retrieval dataset, while the lower section pertains to the particular object image retrieval dataset.

Test Dataset	Number of Database Images	Classes	Query
CUB [51]	5924	100	5924
CARS [52]	8131	98	8131
In-Shop [53]	12,612	3985	14,218
ROxford [56]	4993	13	70
RParis [56]	6322	12	70
Oxford [54,55]	5063	11	55
Paris [55]	6392	11	55

**Table 2:** Performance comparison of the proposed framework with the CLIP-based image retrieval model, UNICOM [6], across three category-level retrieval datasets. Number of Operands is the number of database descriptors from which the distance to the query descriptor is computed. Retrieval Time is the time measured in the online step. Reduction Rate is the percentage decrease compared to UNICOM upon application of the proposed module, while Retention Rate indicates the percentage of maintained accuracy with the proposed framework.

Dataset	Model	Recall@1 (Retention Rate (%))	Number of Operands (Reduction Rate (%))	Retrieval Time (ms) (Reduction Rate (%))
CUB	UNICOM	89.4	5924	3.16
	UNICOM+Ours(Intensive)	87.5 (97.9)	107 (↓ 98.2)	0.17 (↓ 94.6)
	UNICOM+Ours(Auto)	89.2 (99.8)	151 (↓ 97.5)	0.56 (↓ 82.3)
	UNICOM+Ours(Relaxed)	89.5 (100.1)	407 (↓ 93.1)	0.63 (↓ 80.1)
CARS	UNICOM	97.6	8131	4.69
	UNICOM+Ours(Intensive)	96.1 (98.5)	150 (↓ 98.2)	0.14 (↓ 97)
	UNICOM+Ours(Auto)	97.2 (99.6)	228 (↓ 97.2)	0.56 (↓ 88.1)
	UNICOM+Ours(Relaxed)	97.5 (99.9)	457 (↓ 94.4)	0.75 (↓ 84)
In-Shop	UNICOM	86.2	12,612	34.42
	UNICOM+Ours(Intensive)	74.5 (86.4)	219 (↓ 98.3)	0.17 (↓ 99.5)
	UNICOM+Ours(Auto)	80.6 (93.5)	474 (↓ 96.2)	0.73 (↓ 97.9)
	UNICOM+Ours(Relaxed)	85.9 (99.7)	1998 (↓ 84.2)	1.91 (↓ 94.5)

**Table 3:** Performance comparison of the proposed framework with the CNN-based image retrieval model, R-GeM [3], across three particular object retrieval datasets. mAP is mean average precision and the revisited datasets, ROxford and RParis, are categorized into easy, medium, and hard based on the retrieval difficulty, denoted as (E), (M), and (H), respectively.

Dataset	Model	mAP (Retention Rate (%))			Number of Operands	Retrieval Time (ms)
		(E)	(M)	(H)	(Reduction Rate (%))	(Reduction Rate (%))
ROxford	R-GeM R-GeM+Ours(Relaxed)	84.1 83.4 (99.2)	65.3 63 (96.5)	39.9 36.9 (92.5)	4993 1076 (↓ 78.4)	3.26 1.66 (↓ 49.1)
RParis	R-GeM R-GeM+Ours(Relaxed)	91.6 90.8 (99.1)	76.7 76.5 (99.7)	55.3 56.4 (102)	6322 960 (↓ 84.8)	4.56 1.6 (↓ 64.9)
Dataset	Model	mAP (Retention Rate (%))			Number of Operands (Reduction Rate (%))	Retrieval Time (ms) (Reduction Rate (%))
Oxford	R-GeM R-GeM+Ours(Relaxed)	88.2 82.9 (94)			5063 1112 (↓ 78)	3.47 1.7 (↓ 54.1)
Paris	R-GeM R-GeM+Ours(Relaxed)	92.6 92.2 (99.6)			6392 988 (↓ 84.5)	5.75 1.62 (↓ 71.8)

## Data Availability

Data are contained within the article.
